# The effects of carvacrol and p‐cymene on Aβ_1‐42_‐induced long‐term potentiation deficit in male rats

**DOI:** 10.1111/cns.14459

**Published:** 2023-09-19

**Authors:** Sahifeh Kazemi, Samaneh Safari, Somayeh Komaki, Seyed Asaad Karimi, Zoleikha Golipoor, Alireza Komaki

**Affiliations:** ^1^ Department of Neuroscience, School of Science and Advanced Technologies in Medicine Hamadan University of Medical Sciences Hamadan Iran; ^2^ Student Research Committee Hamadan University of Medical Sciences Hamadan Iran; ^3^ Department of Physiology, School of Medicine Hamadan University of Medical Sciences Hamadan Iran; ^4^ Cellular and Molecular Research Center, Faculty of Medicine Guilan University of Medical Sciences Rasht Iran

**Keywords:** Alzheimer's disease, carvacrol, long‐term potentiation; hippocampus, p‐cymene, synaptic plasticity, β‐amyloid

## Abstract

**Aims:**

Alzheimer's disease (AD) is the most common type of dementia in which oxidative stress plays an important role. In this disease, learning and memory and the cellular mechanism associated with it, long‐term potentiation (LTP), are impaired. Considering the beneficial effects of carvacrol (CAR) and p‐cymene against AD, their effect was assessed on in vivo hippocampal LTP in the perforant pathway (PP)‐dentate gyrus (DG) pathway in an Aβ_1‐42_‐induced rat model of AD.

**Methods:**

Male Wistar rats were randomly assigned to five groups: sham: intracerebroventricular (ICV) injection of phosphate‐buffered saline, Aβ: ICV Aβ_1‐42_ injections, Aβ + CAR (50 mg/kg), Aβ + p‐cymene (50 mg/kg), and Aβ + CAR + p‐cymene. Administration of CAR and p‐cymene was done by gavage daily 4 weeks before and 4 weeks after the Aβ injection. The population spike (PS) amplitude and field excitatory postsynaptic potentials (fEPSP) slope were determined in DG against the applied stimulation to the PP.

**Results:**

Aβ‐treated rats exhibited impaired LTP induction in the PP‐DG synapses, resulting in significant reduction in both fEPSP slope and PS amplitude compared to the sham animals. Aβ‐treated rats consumed either CAR or p‐cymene separately (but not their combination), and showed an enhancement in fEPSP slope and PS amplitude of the DG granular cells.

**Conclusions:**

These data indicate that CAR or p‐cymene can ameliorate Aβ‐associated changes in synaptic plasticity. Surprisingly, the combination of CAR and p‐cymene did not yield the same effect, suggesting a potential interaction between the two substances.

## Graphical abstract

We investigated the effect of the treatment with carvacrol (CAR) and p‐cymene on in vivo hippocampal long‐term potentiation (LTP) in the perforant pathway‐dentate gyrus pathway in the Aβ1‐42‐induced rat model of Alzheimer's disease. Treatment with the CAR or p‐cymene alone can prevent synaptic plasticity impairment caused by Aβ1‐42. Interestingly, our results showed that the combined treatment with CAR and p‐cymene could not prevent the destructive effects of Aβ on hippocampal LTP. Further investigations are needed to determine the detailed mechanism(s) of action of CAR or p‐cymene.

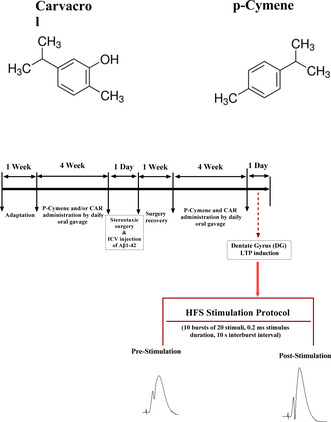

## 1 INTRODUCTION

Dementia is characterized by a decline in thinking, memory, behavior, cognitive function, calculation, orientation, comprehension, language, learning capacity, and judgment, and the capability to do daily activities.^1^ Dementia almost affects the elderly, but it is not a normal part of aging. About 50 million people suffer from dementia worldwide with about 10 million new cases annually.^2^ The physical, social, psychological, and economic effects of dementia have been declared to affect the patient and his career, families, and society.^3^ Alzheimer's disease (AD) has been known as the commonest form of dementia accounting for 60%–70% of cases.^4^

The accumulation of beta‐amyloid (Aβ) plaques and tau tangles: neurofibrillary tangles (NFT) are some brain alterations associated with AD.^5^ Lack of cholinergic and adrenergic function,^6^ oxidative stress,^7^ inflammation,^8^ steroid hormone deficiencies,^9^ and excitotoxicity^10^ have been suggested as the other mechanisms for developing AD.

Synaptic pathology and changes in synaptic plasticity in the brain have been proposed as possible early markers of both AD and aging.^11^, ^12^, ^13^ Synaptic plasticity occurs locally in individual synapses and consists of long‐term potentiation (LTP) or long‐term depression (LTD).^14^, ^15^ AD is associated with the suppression of LTP and an elevation of LTD in the hippocampus.^13^, ^16^, ^17^ Aβ_1–42_ is the major mediator of the cognitive impairments in AD.^17^

Some drugs and therapeutic techniques can temporarily control AD symptoms, but there is no permanent treatment. Therefore, the identification of novel therapeutic candidates to slow down or inhibit AD progression is highly important.^18^

None of the available medications for AD stop neuronal damage and destruction leading to AD symptoms and mortality. Two classes of drugs have been approved for the treatment of AD, including acetylcholinesterase inhibitors (donepezil, rivastigmine, and galantamine) and memantine as the NMDA receptor antagonist. Despite significant advances in understanding AD pathogenesis, current therapies address only moderate signs of impaired brain function^19^, ^20^ there is no effective treatment to control AD‐related hippocampal‐synaptic plasticity impairments. Here, we examined the effect of carvacrol (CAR) (5‐isopropyl‐2‐methyl phenol) and p‐cymene on Aβ‐induced LTP deficit in male rats. CAR and p‐cymene were found with therapeutic potential in preventing or modulating AD.

CAR is a phenolic monoterpenoid that is present in the essential oil of some aromatic plants, like species of *Zataria*, *Origanum*, *Thymbra*, *Thymus*, *Satureja*, *Lepidium flavum*, *Citrus uranium Bergama*, and *Coridothymus* belonging to the family *Lamiaceae* and *Lippia* of Verbenaceae.^21^, ^22^ The effectiveness and safety of CAR to alleviate cognitive deficits due to an increase in Aβ levels or cholinergic hypofunction in rats have been declared.^23^ Also, it has been reported that CAR attenuates cytotoxicity induced by Aβ via activating protein kinase C (PKC) and inhibiting oxidative stress in PC12 cells.^24^ CAR has antimicrobial, antioxidant, anti‐cancer, and anti‐inflammatory activities.^22^, ^25^ CAR has also been reported to act as an inhibitor of acetylcholinesterase.^26^, ^27^

p‐Cymene as a naturally occurring aromatic organic compound is classified as an alkylbenzene because of a monoterpene. It is found in essential oils, such as cumin and thyme oils. It is employed as a prominent mediator in medications as well as a flavoring compound.^28^ It improves AD‐induced disorders, such as memory impairment through antioxidant and anti‐inflammatory properties and also direct anti‐fibril effect.^29^ Also, it has been shown that p‐cymene acts as an inhibitor of Aβ peptide aggregation and Aβ‐induced cytotoxicity.^30^

**FIGURE 1:**
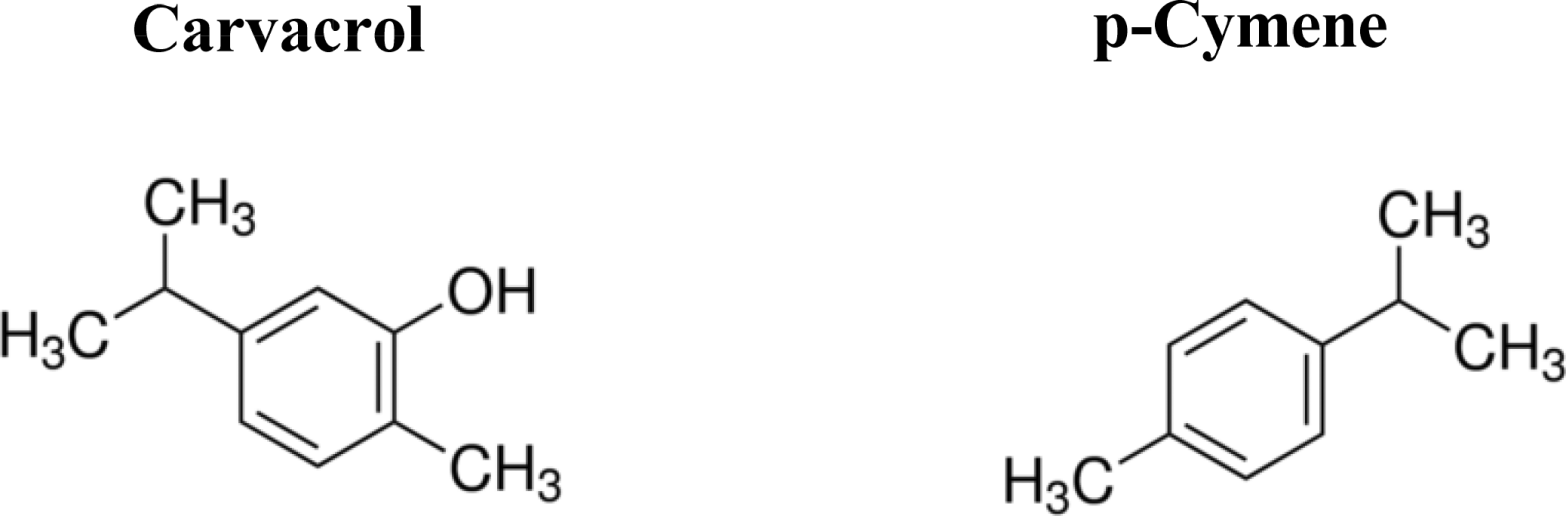
Chemical structure of the carvacrol and p‐cymene.

The novelty of this work lies in the investigation of the effects of CAR and p‐cymene on Aβ‐induced LTP deficit in male rats. While the effect of CAR and p‐cymene in alleviating cognitive deficits due to increased Aβ levels have been declared before, there has been no existing data on their specific impact on LTP on the Aβ‐induced LTP impairment. Understanding how these compounds may affect LTP, a crucial neural mechanism underlying memory and learning processes, is important. Considering the beneficial effects of CAR and p‐cymene (Figure 1), here, we assessed their effect on in vivo hippocampal LTP in the perforant pathway (PP)‐dentate gyrus (DG) pathway in an Aβ_1‐42_ ‐induced rat model of AD.

## 2 MATERIALS AND METHODS

### 2.1 Ethics statement

The experiments using rats were performed following the animal care and use guidelines confirmed by the Institutional Ethics Committee (IR.UMSHA.REC.1394.582), Hamadan University of Medical Sciences, and according to the National Institutes of Health Guide for Care and Use of Laboratory Animals.^31^ Minimizing the animal suffering was considered. Experiments leading to pain and distress were conducted in another room where other animals were not present.

### 2.2 Animals and experimental design

**FIGURE 2:**
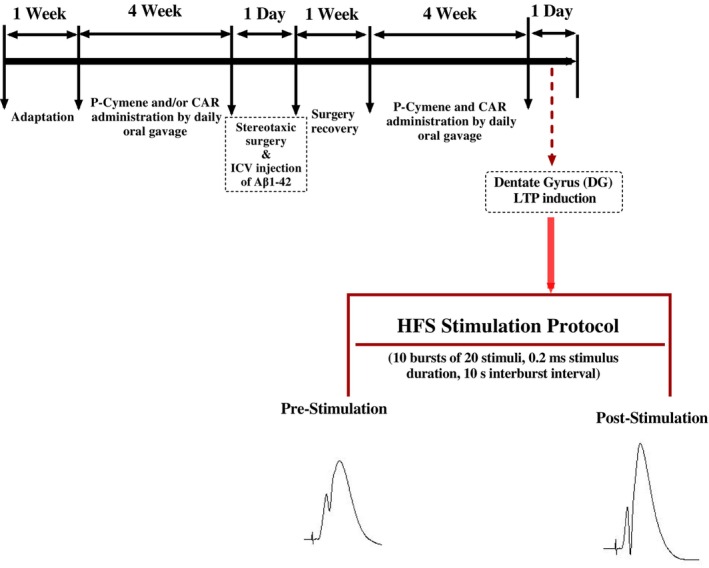
Following 1 week of adaptation, the rats received carvacrol (CAR) and/or p‐cymene via oral gavage 4 weeks before and 4 weeks after the ICV Aβ_1‐42_ injection. Then, after anesthetization with urethane, animals were positioned in a stereotaxic device for surgery, implantation of the electrode, and field potential recording. After observing a stable baseline for at least 40 min, LTP induction was done with high‐frequency stimulation (HFS) (10 bursts of 20 stimuli, 0.2 ms stimulus duration, 10‐s interburst interval) in the dentate gyrus (DG) of rats.

Male Wistar rats (200–250 g body weight) provided by the animal breeding colony of Hamadan University of Medical Sciences were kept at 22 ± 2°C under a 12/12‐h light/dark cycle (light on 7 a.m.) with free access to food and water. The rats were kept in cages with 2–3 animals per cage. One week of adaptation was considered and then, the rats were randomly assigned to the following groups (*n* = 6–8 per group): group 1 (sham; intracerebroventricular [ICV] injection of phosphate‐buffered saline [PBS] into the right lateral ventricle and administration of saline through gavage once daily, starting 4 weeks before and 4 weeks after the ICV injection), group 2 (Aβ; ICV injections of Aβ_1‐42_ and administration of saline through gavage once daily, starting 4 weeks before and 4 weeks after the ICV injection), group 3 (Aβ + CAR; 50 mg/kg of CAR was administered via oral gavage, once a day, for 4 weeks before and 4 weeks after the Aβ injection), group 4 (Aβ + p‐cymene; 50 mg/kg of p‐cymene was administered through gavage, once a day 4 weeks before and 4 weeks after the Aβ injection), group 5 (Aβ + CAR + p‐cymene; 50 mg/kg of CAR and 50 mg/kg of p‐cymene was administered through gavage, once a day, 4 weeks before and 4 weeks after the Aβ injection). Doses of CAR^32^, ^33^ and p‐cymene^29^, ^34^ were selected based on the previous reports. Two months later, LTP was induced in DG using high‐frequency stimulation (HFS). Figure 2 displays the experimental design and timeline.

### 2.3 ICV injection of Aβ_1‐42_ and neurosurgical procedure

Aβ_1‐42_ (1 mg; Tocris Bioscience Bristol) dissolved in 1 mL of PBS (vehicle) followed by incubation (7 days/37°C) before usage that led to the formation of Aβ fibrils (with neurotoxic activity).^35^, ^36^ Animals were anesthetized by i.p. injection of ketamine (100 mg/kg) and xylazine (10 mg/kg) and transferred to the stereotaxic device (Stoelting Co.).^37^ The skull was exposed over the ventricular area based on the following coordinates: 2 mm lateral to the midline, 1.2 mm posterior to bregma, and 4 mm ventral to the surface of the cortex.^38^ The Hamilton microsyringe (5 μL; USA) was employed for injections within 5 min and the injections were done gently (1 μL/min) into the right lateral ventricle. After injection, the syringe was kept in the injection area for 5 min and then gently removed. Animals had a recovery time of 7 days.^39^

### 2.4 The surgical procedure, electrophysiological recording, and LTP induction

**FIGURE 3:**
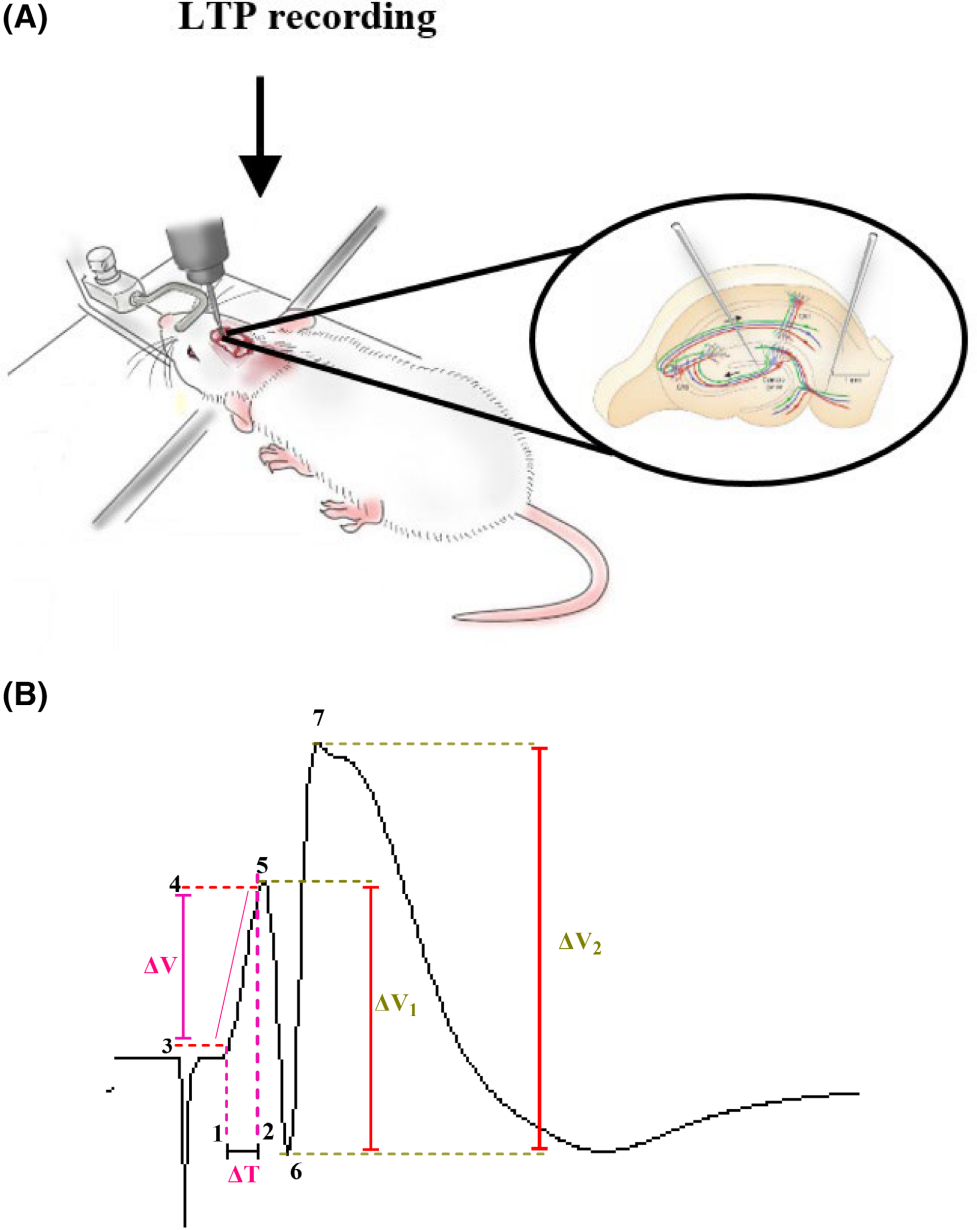
Recording of LTP (A) and measurement of PS amplitude and EPSP slope (B). PS amplitude and EPSP slope were determined using Equations 1 and 2, respectively (see the text). ΔT, time difference and ΔV, the potential difference.

CAR and p‐cymene were administered intragastrically by gavage daily 4 weeks before and 4 weeks after the Aβ injection. Then, after anesthetization with urethane, animals were placed in the stereotaxic device for surgery, implantation of the electrode, and field potential recording (Figure 3A). The used procedures were similar to our previous studies.^40^, ^41^, ^42^, ^43^, ^44^, ^45^ In brief, under urethane anesthesia (intraperitoneal injection, 1.5 g/kg), the rat's head was fixed in the stereotaxic device for surgical procedure and electrophysiological recording. Animals' body temperature was kept at 36.5 ± 0.5°C using a heating pad. After drilling small holes in the skull, two stainless steel bipolar electrodes (125 μm diameter, Advent Co.) covered by Teflon were positioned in the right cerebral hemisphere. The stimulating electrode was located in the PP (: AP:−8.1 mm from bregma; ML: +4.3 mm from midline; DV: 3.2 mm from the skull surface), whereas the recording electrode was located in the DG granular cell layer (AP:−3.8 mm from bregma; ML: +2.3 mm from midline; DV: 2.7–3.2 mm from the skull surface) based on the Paxinos and Watson atlas.^38^, ^42^, ^46^ For minimizing trauma to the brain tissue, electrodes were lowered very gently (0.2 mm/min) from the cortex to the hippocampus.

**FIGURE 4:**
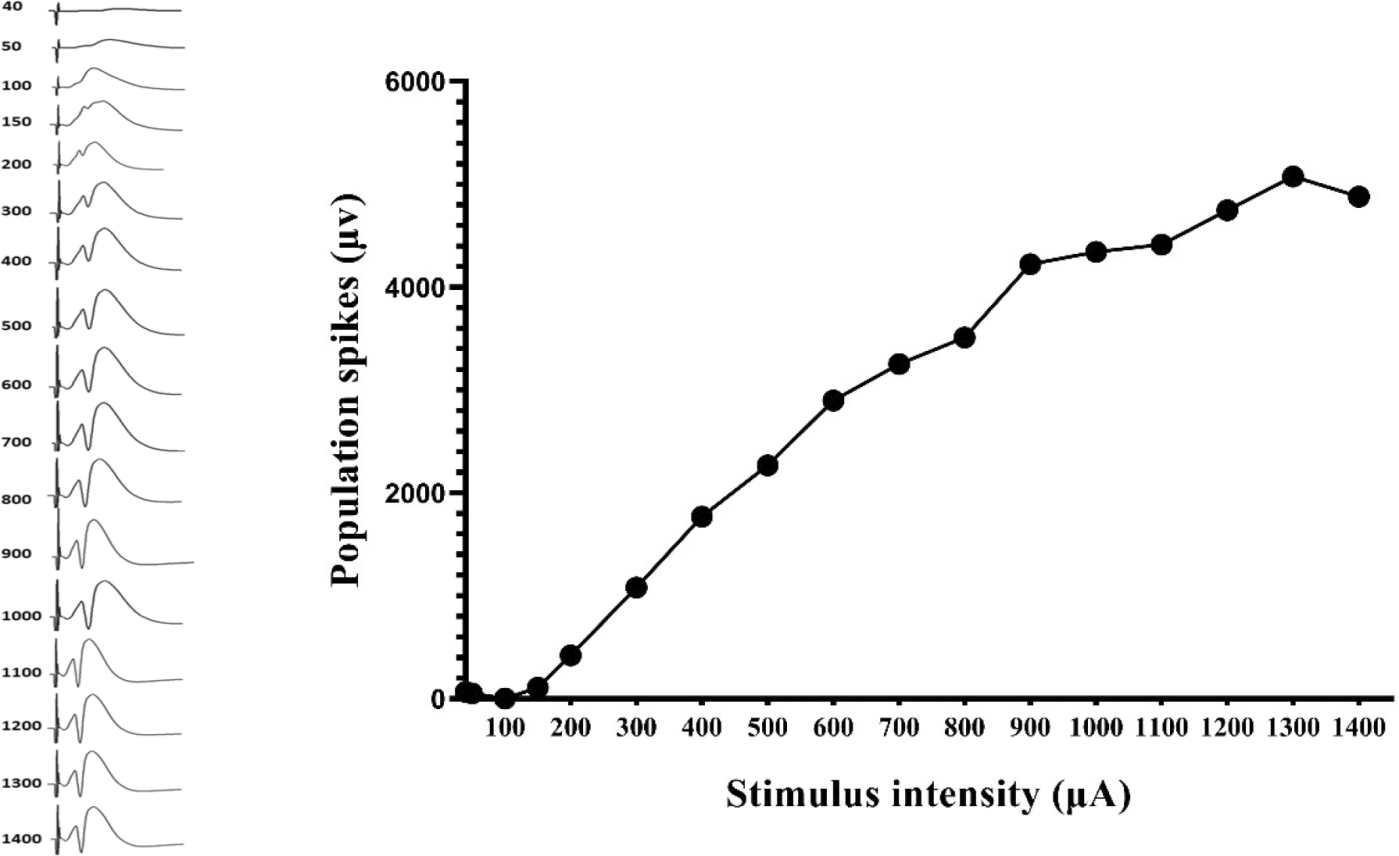
Input‐output current profiles were achieved through the stimulation of the PP for determining the stimulus intensity for each rat.

Input‐output current profiles were achieved through the stimulation of the PP for determining the stimulus intensity for each rat (80% maximal population spike) (Figure 4). Single biphasic square‐wave pulses (0.1 ms) were delivered by constant current isolation units (A365 WPI) at a frequency of 0.1 Hz.

The field potential recordings were obtained in the DG granular cells after stimulating the PP. The PP received test stimuli every 10 s. Electrodes were located for eliciting the maximal amplitude of population spike (PS) and field excitatory postsynaptic potentials (fEPSP). After ensuring a steady‐state baseline response that lasted about 40 min, LTP induction was done using an HFS protocol of 400 Hz (0.2 ms stimulus duration, 10 bursts of 20 stimuli, 10‐s interburst interval) at an intensity, which could evoke a PS amplitude and field EPSP slope of approximately 80% of the maximum response. The fEPSP and PS were recorded 5, 30, and 60 min following the HFS for determining the alterations in the synaptic response of DG neurons. For each time point, 10 continuous evoked responses were averaged at a stimulus interval of 10 s.^47^, ^48^, ^49^, ^50^

The stimuli parameters were defined in homemade software followed by sending through a data acquisition board connected to a constant current isolator unit (A365 WPI) before delivery to the PP using the stimulus electrodes. A preamplifier was employed to pass the induced field potential response from the DG and followed by amplification (1000×) (Differential amplifier DAM 80 WPI), and filtration (bandpass 1 Hz to 3 kHz). The response was digitized at 10 kHz and observed on a computer (and an oscilloscope). It was then saved in a file for the next offline analyses.

### 2.5 Measurement of evoked potentials

PS and fEPSP are two components of the evoked field potential in the DG. During electrophysiological recordings, alterations in PS amplitude and fEPSP slope were examined.^42^

Equations 1 and 2 were used to calculate PS amplitude and EPSP slope, respectively (Figure 3B).

(1) $$\text{EPSP}=\frac{\mathrm{\Delta V}}{\mathrm{\Delta T}}$$

(2) $$\mathrm{PS}=\frac{\Delta V_{1}+\Delta V_{2}}{2}$$

ΔV = the potential difference between points 3 and 4.

ΔT: time difference between points 1 and 2.

ΔV1 = the potential difference between points 5 and 6.

ΔV2 = the potential difference between points 6 and 7.

### 2.6 Statistical analysis

Data are expressed as mean ± SEM and analyzed by GraphPad Prism® 8.0.2 software. The Shapiro‐Wilk test checked the normal distribution of the data. LTP data were analyzed through two‐way repeated‐measures ANOVA followed by the Bonferroni test. LTP data were normalized to the mean value of fEPSP slopes and PS amplitude recorded before LTP induction (Equation 3).^43^, ^51^, ^52^ *p*‐values less than 0.05 were regarded as significant.

(3) $$\mathrm{LTP}=\frac{\text{the EPSP or}\;\mathrm{PS}\;\text{value after}\;\mathrm{HFS}\;\text{induction}\times 100\%}{\text{the average EPSP or}\;\mathrm{PS}\;\mathrm{at}\;\text{baseline}}$$

## 3 RESULTS

### 3.1 Effects of CAR and p‐cymene on the fEPSP slopes of DG granular cells of Aβ‐treated rats

**FIGURE 5:**
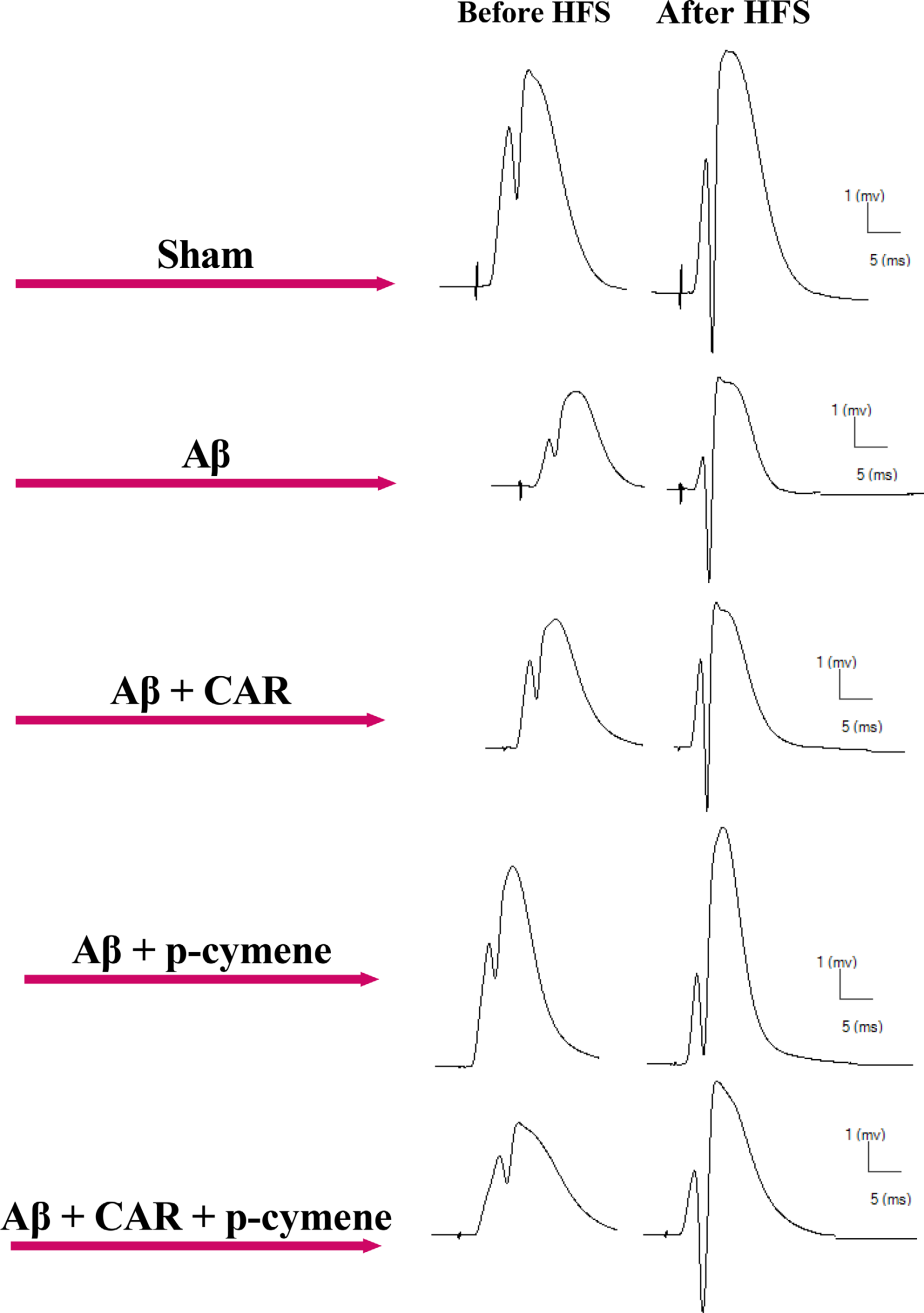
Representative sample traces of evoked field potential in the dentate gyrus were recorded before and 60 min following the high‐frequency stimulation in all groups.

**FIGURE 6:**
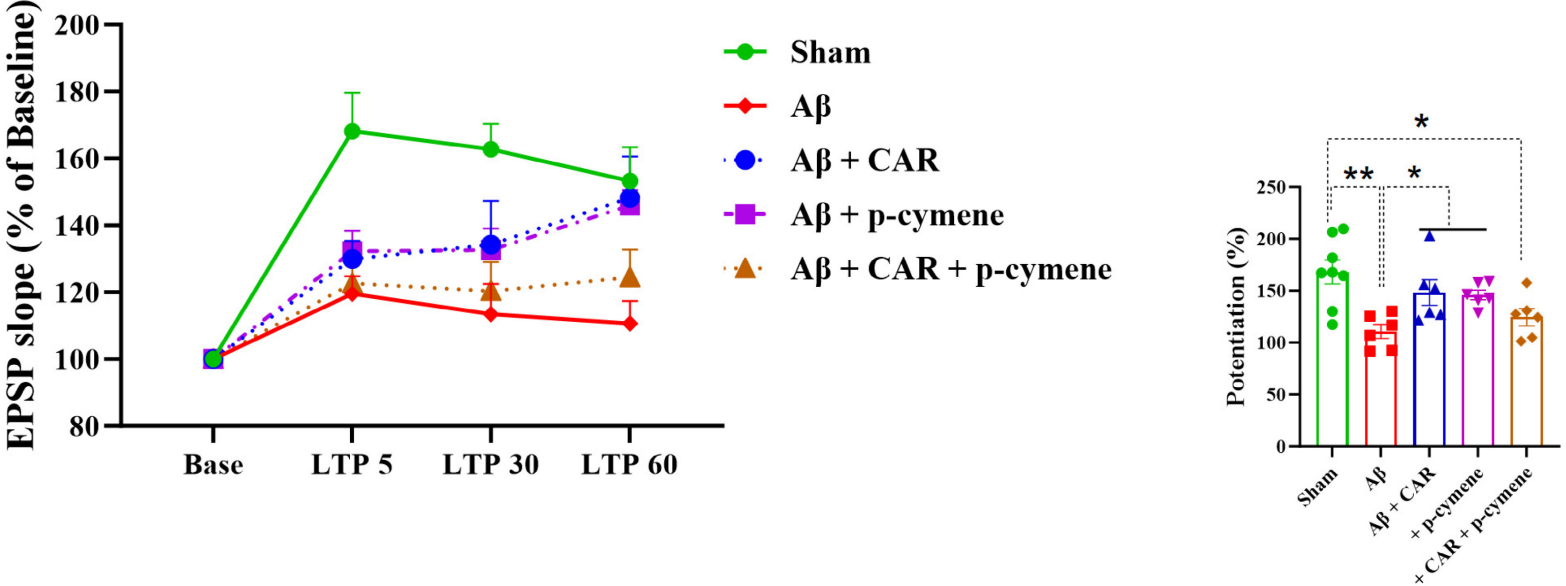
Time‐dependent alterations in hippocampal responses against perforant path stimulation after high‐frequency stimulation (HFS). The groups were found with significantly different long‐term potentiation (LTP) of the EPSP slope in dentate gyrus (DG) granular cell synapses of the hippocampus. The left panel displays changes (%) in the fEPSP slope versus time after HFS in all groups. Bar graphs display the average fEPSP slope changes (%) within 60 min after HFS. Treatment with carvacrol (CAR) and p‐cymene (but not their combination) prevented Aβ‐induced impairment of LTP expressed as the slope of fEPSP in the DG. Data are expressed as mean ± SEM % of baseline. *p < 0.05 and **p < 0.01.

**FIGURE 7:**
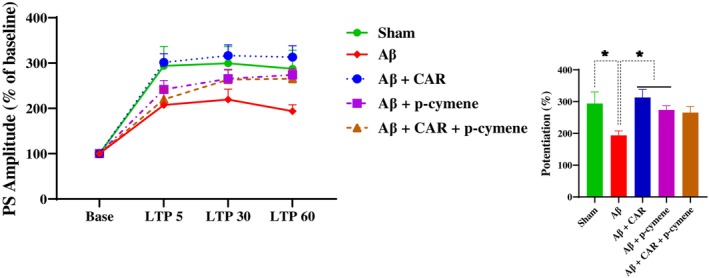
Time‐dependent alterations in hippocampal responses against perforant path stimulation after high‐frequency stimulation (HFS). The groups were found with significantly different long‐term potentiation (LTP) of the PS amplitude in dentate gyrus (DG) granular cell synapses of the hippocampus. The left panel displays PS amplitude changes (%) versus time after HFS in all groups. Bar graphs display the average PS amplitude changes (%) within 60 min after HFS. Treatment with carvacrol (CAR) and p‐cymene (but not their combination) prevented Aβ‐induced impairment of LTP expressed as the slope of the fEPSP in the DG. Data are expressed as mean ± SEM % of baseline. *p < 0.05.

Field potential recordings were obtained in the DG granular cells after PP stimulation. Figure 5 displays a representative example of evoked field potential in the DG recorded before and 60 min following HFS. The effects of CAR and p‐cymene on the EPSP slopes as well as PS amplitudes of Aβ‐treated rats are illustrated in Figures 6 and 7, respectively. HFS did not induce LTP in Aβ‐treated rats (*F* [3, 20] = 1.740, *p* = 0.1911, one‐way ANOVA). The one‐way ANOVA was used to test whether there were significant differences before and after LTP induction (at different time points). Aβ‐treated rats were found with a significant decrease in changes in the slope of fEPSP immediately and 60 min following HFS than the sham group. A two‐way ANOVA was used to indicate the differences between the groups. A significant effect of time (*F* [2.463, 66.50] = 43.32, *p* < 0.0001) and treatment (*F* [4, 27] = 6.197, *p* = 0.0011) in the slope of EPSP of the granular cell of DG was observed (Figure 6). The post‐hoc analysis revealed a significant difference between the sham and Aβ‐treated groups. EPSP Slope was reduced in the Aβ‐treated rats compared with the sham group (*p* = 0.0015; Figure 6). CAR or p‐cymene consumption by the Aβ‐treated rats increased the EPSP slope of the DG granular cells (*p* < 0.05; Figure 6). The changes in the fEPSP slope immediately and 60 min following HFS were significantly greater in the Aβ + CAR and Aβ + p‐cymene groups than in the Aβ‐treated rats.

### 3.2 Effects of CAR and p‐cymene on the PS amplitude of DG granular cells of Aβ‐treated rats

A significant effect of time (*F* [3, 120] = 64.80, *p* < 0.0001) and treatment (*F* [4, 120] = 6.510, *p* < 0.0001) on PS amplitude of the DG granular cells was observed (Figure 7). The post‐hoc analysis revealed a significant difference between the sham and Aβ‐treated groups (*p* < 0. 05; Figure 6). PS amplitude was reduced in the Aβ‐treated rats compared with the sham group. CAR and p‐cymene consumption by the Aβ‐treated rats increased the PS amplitude of the DG granular cells (*p* < 0.05; Figure 7).

## 4 DISCUSSION

We investigated the effect of the treatment with CAR and p‐cymene on in vivo hippocampal LTP in the PP‐DG pathway in the Aβ_1‐42_‐induced rat model of AD. Aβ impaired LTP induction in the PP‐DG synapses. This observation is confirmed by a decrease in EPSP slope and PS amplitude of LTP. Therefore, the observations of previous studies, in which Aβ caused synaptic plasticity impairment was confirmed. In the present study, it was for the first time observed that treatment with CAR or p‐cymene alone can prevent the destructive effects caused by Aβ on hippocampal synaptic plasticity. Interestingly, our results showed that the combined treatment with CAR and p‐cymene could not prevent the Aβ destructive effects on hippocampal LTP.

Our results indicated that CAR and p‐cymene attenuated LTP deficit induced by Aβ_1‐42_. The underlying mechanisms of the effectiveness of CAR in rat models of dementia can be owing to its anticholinesterase, antioxidant, anti‐apoptotic, and anti‐inflammatory activities.^23^, ^53^, ^54^, ^55^ CAR has been shown to decrease the expression of caspase‐3.^56^ Caspases are involved in apoptosis and also play a role in the development of AD.^57^, ^58^ They can be activated upon exposure to Aβ.^59^ Recent evidence suggests that Caspase‐3 activation by mitochondria is needed for hippocampal synaptic plasticity.^60^ Moreover, it has been reported that Aβ_1‐42_‐induced inhibition of LTP is mediated by a signaling pathway involving caspase‐3, Akt1, and glycogen synthase kinase‐3 beta (GSK‐3β)^17^ in rats and mice, where caspase‐3 is needed for the suppression of LTP by Aβ. Accordingly, it can be concluded that CAR may prevent the destructive effects of Aβ on hippocampal LTP by reducing caspase‐3 expression. Also, Aβ_1‐42_ leads to a sequential decrease in the levels of phosphorylated‐Akt (also known as protein kinase B, PKB),^61^ and treatment with CAR increases the levels of phosphorylated Akt.^56^ PI3K/Akt is involved in LTP induction in the PP‐DG synapses.^62^

PKC is also critical for the induction of LTP.^63^ Signaling deficits of the PKC pathway are involved in the pathophysiology of AD.^64^, ^65^ CAR has been shown to have a stimulatory effect on PKC activity.^24^ PKC activation can prevent synaptic loss, an elevation in Aβ, and cognitive impairments in AD mice.^66^ In addition, CAR increases the expression of proteins associated with neuronal and synaptic plasticity (F‐actin, β‐tubulin III, GAP‐43, 200‐kDa neurofilament, and synapsin‐I) and improves bioenergy sensing (p‐AMPKα, AMPKα, and ATP).^55^ The neuroprotection effects of CAR have also been attributed to its ability to block the transient receptor potential, inhibit neuronal NOS, and regulate Ca^2+^ homeostasis.^67^ Therefore, it seems that these mechanisms may be associated with the results obtained in the current study; however, there is a need for more investigations in the future.

p‐cymene possesses antioxidant, anti‐inflammatory, and direct anti‐fibril effects,^29^ and can inhibit Aβ aggregation and Aβ‐induced cytotoxicity.^30^ Inflammation has been shown to play a major role in AD pathogenesis^68^ and the p38 MAPK pathway plays a key role in this regard.^69^ There is an association between p38 MAPK pathway immunoreactivity and neurotic Aβ plaques and neurofibrillary tangle‐bearing neurons.^69^ The p38 mitogen‐activated protein kinase (MAPK or MAP kinase) cascade is a signal transducer downstream of LTP induction (e.g., NMDA or metabotropic glutamate receptors activation). Limited information is available regarding the importance of p38 MAPK in the synaptic plasticity alternation caused by AD pathophysiology; however, it has been shown that p38 MAPK activation is associated with memory impairment in AD. Origlia et al. indicated that Aβ impairs LTP in the entorhinal cortex by neuronal receptor for advanced glycation end products (RAGE)‐mediated activation of p38 MAPK.^70^ Therefore, p38 MAPK activation is needed for LTP impairment after Aβ exposure. In addition, p‐cymene has been shown to block activation of the MAPK signaling pathway,^71^ which possibly ameliorates the destructive effects of Aβ on hippocampal LTP.

Aβ can inhibit hippocampal LTP through tumor necrosis factor receptor/IKK/NF‐κB pathway.^72^ NF‐κB activity is associated with the pathogenesis of AD and can be considered as a candidate for the treatment of AD.^73^, ^74^ Inhibition of NF‐κB activity reduces Aβ levels in the AD.^75^ Also, *p‐cymene* reduced MAPK and NF‐κB activity as well as TNF‐α production.^76^ Inhibition of LTP by TNF‐α has been reported in the literature,^77^ and p‐cymene can prevent the destructive effects of Aβ on hippocampal LTP by reducing TNF‐α production.

Finally, it was interesting to observe that the combined treatment with CAR and p‐cymene could not prevent the destructive effects of Aβ on hippocampal LTP, which may be due to the extensive elimination of reactive oxygen species (ROS). ROS act as signaling molecules and regulate many physiological processes.^78^, ^79^ They also cause reversible post‐translational protein changes for regulating signaling pathways.^78^ ROS at normal levels play a role in mediating several cellular responses, such as cell growth and immunity.^80^ ROS generation is caused by basic metabolic processes; nonetheless, high levels of ROS result in DNA damage, lipid peroxidation, and even cell death.^81^, ^82^ CAR and p‐cymene have antioxidant activity. However, the imbalance of the excessive antioxidant capacity (antioxidative stress) is just as harmful as oxidative stress. Despite destructive effects, the consequences of oxidative stress may be beneficial for several physiological processes in cells. On the other hand, “antioxidative stress,” particularly in the cases of overconsumption of synthetic antioxidants is associated with destructive effects.^83^ The combined treatment with CAR and p‐cymene may lead to antioxidant stress that failed to counteract the destructive effects of Aβ on hippocampal LTP. It is possible that, despite individually demonstrating potential therapeutic benefits, the combination of CAR and p‐cymene did not produce a synergistic effect and may have even interacted in a way that reduced their overall effectiveness. Additionally, the timing of treatment may have been suboptimal, as the compounds may have needed to be administered earlier or later in the disease progression to have a positive impact. Further studies are needed in this regard.

## 5 CONCLUSION

In summary, treatment with the CAR or p‐cymene alone can prevent synaptic plasticity impairment caused by Aβ_1‐42_. Interestingly, our results showed that the combined treatment with CAR and p‐cymene could not prevent the destructive effects of Aβ on hippocampal LTP. Further investigations are needed to determine the detailed mechanism (s) of action of *CAR* or *p‐cymene*.

## AUTHOR CONTRIBUTIONS

Alireza Komaki, Sahifeh Kazemi, and Samaneh Safari contributed to the study design, statistical analysis of data, writing, and critical revision of the manuscript; Seyed Asaad Karimi, Somayeh Komaki, and Zoleikha Golipoor conducted experiments, data acquisition, and drafting of the manuscript; Samaneh Safari, Seyed Asaad Karimi, and Alireza Komaki were responsible for administrative, technical, and material support and critical revision of the manuscript for important intellectual content; Alireza Komaki supervised the study. All authors read and approved the final manuscript.

## FUNDING INFORMATION

This study was funded by a grant (Grant No: 14020219911) of the Hamadan University of Medical Sciences, Hamadan, Iran.

## CONFLICT OF INTEREST STATEMENT

There are no conflicts of interest to declare.

## Data Availability

The datasets generated and/or analyzed during this study are available from the corresponding author on reasonable request.
